# Book Reviews

**Published:** 1894-12

**Authors:** 


### Book Reviews.

A SYSTEM OF GENITO-URINARY DISEASES, Syphilology and Dermatology. By various authors. Edited by PRINCE A. MORROW, A. M., M. D., Clinical Professor of Genito-Urinary Diseases; formerly Lecturer on Dermatology in the University of the City of New York; Surgeon to the Charity Hospital. etc. With illustrations. In three large 8vo volumes. Volume III. Dermatology. Pp. xiv.—976. New York: D. Appleton & Co. 1893. Sold only by subscription.

Modern methods of investigation have served to differentiate and enlarge the catalogue of diseases in almost all departments of medicine. This is especially true in dermatology, as may be observed by an examination of this treatise, wherein are recorded some forty or more diseases that a few years ago were unknown or were blended with other affections of the skin.

This work is divided into two parts. In Part I., called general, are treated the anatomy and physiology of the skin by Louis Heitzmann; semeiology, by Prince A. Morrow; general etiology, by William A. Hardaway, and classification by Prince A. Morrow.

In Part II., classified as special, the various diseases of the skin are treated upon. These special diseases are divided into classes, and in the first class come inflammations. Here we find the exanthemata and every physician should familiarize himself with this portion of the subject, since general practitioners of medicine are always called upon to treat scarlet fever, measles, small-pox and the like.

In this class also are found erythemata, erysipelas, furunculus, herpes, pemphigus, the dermatites, etc., which are interestingly handled by various authors.

The second class includes hemorrhages and in which purpura simplex and purpura hemorrhagica are briefly considered.

The third class embraces the hypertrophies, and in which, besides many other diseases, are considered elephantiasis, acromegaly and myxedema. The fourth class includes the atrophies, leucoderma, albinism and vitiligo.

New growths are grouped in the fifth class, where interesting and profitable studies may be made of the several tumors of the skin. Perhaps one of the most interesting chapters here is the one on leprosy, prepared by the editor, largely from personal researches. Much interest attaches to the history of Father Damien's own case, which is here given, together with a photograph of this self-sacrificing priest, taken a month before his death.

The sixth class embraces the neuroses in which pruritus is included, while in class seven diseases of the appendages of the skin are included; sweat glands, sebaceous glands, hair follicles and nails.

Finally, in class eight are grouped parasitic diseases, both vegetable and animal. The work is handsomely printed and well illustrated. It is the most important contribution to the literature of dermatology that has been made, and is a guide for the general practitioner and specialist.

This is the third and final volume in the system of genito-urinary diseases, syphilology and dermatology edited by Dr. Morrow, and published by D. Appleton & Co.

---

THE JOHNS HOPKINS HOSPITAL REPORTS. Volume IV., Nos. 1, 2, 3. Report on Typhoid Fever. Quarto, pp. 167. Baltimore: The Johns Hopkins Press. 1894.

The management of typhoid fever has undergone considerable change within the last few years. Formerly it was supposed that the administration of drugs was a potent factor in the cure of this disease. Now it is generally believed by the most experienced clinicians that, as a rule, the fewer drugs administered the better.

This change of front does not belittle the offices of the physician in his ministrations to typhoid patients. On the contrary, it magnifies them and makes them more exalted. The mere administration of drugs to combat symptoms may be reduced to a simple question of mechanics that any tyro may acquire in a few weeks; whereas, to safely conduct a typhoid case through the varying stages of the disease, under the present plan of nursing, diet and baths, requires an exercise of judgment which can only be obtained by experience, and is akin to that surgical judgment which is apart from mere operative technique, but tells the surgeon when and where to operate and also when to withhold the knife.

Osler informs us in this work that about 75 per cent. of typhoid patients recover under any and all forms of treatment, even without good nursing and regulated diet; but, he adds, that by judicious care, careful feeding and by the withholding of drugs of uncertain value, fifteen additional patients in each hundred are saved. Add to this, according to statistics, the 3 or 4 per cent. that are saved by hydrotherapy and we have a mortality reduced to 6 or 7 per cent.

The essentials, then, in the successful management of typhoid fever are good nursing, carefully regulated diet, fresh air, the cold bath and a minimum of medicines. Not everyone, to be sure, can endure the immersion bath, but the cold bath is applicable to most cases in some of its modifications.

When the profession of medicine comes to believe with Osler, that drugs, as a rule, are not indicated in the management of typhoid fever; that no known drug shortens by a day the course of the fever; that no method of specific treatment or of antisepsis of the bowel can be relied upon, and especially, that the so-called antipyretics of the coal-tar series are dangerous and often cause death through the production of cardiac failure, the management of this disease will have advanced to an intelligent stage in which the mortality will not be greater than 3 per cent.

Nothing but good can come of a universal reading of this work.

---

A SYSTEM OF LEGAL MEDICINE. By ALLAN McLANE HAMILTON, M. D., Consulting Physician to the Insane Asylums of New York City, etc., etc., and Lawrence Godkin, Esq., of the New York Bar. With twenty-seven collaborators. Illustrated. Volume I. Large 8vo, pp. 657. Price per volume, cloth, $5.50 ; full sheep, $6.50. New York : E. B. Treat, 5 Cooper Union. 1894.

The list of contributions to this work includes the names of many distinguished writers and authorities upon medical jurisprudence in this country.

For many years the contributions to this subject have been sparse, though Beck's treatise, now nearly obsolete, was, when issued, one of the greatest in any country and became a text-book in all the schools.

Hamilton has wisely planned his book, and it deals with questions that possess lively interest at present. It may be asserted that there is a revival of interest in medical jurisprudence among American physicians. They are nowadays so frequently called into the courts as medical experts, even in the smaller towns, that it behooves each and every one to qualify himself for the ordeal. There is no more pitiable spectacle than the discomfiture of a medical witness at the hands of a keen and unscrupulous lawyer.

Hamilton's work begins at the right end of the lever. After a short introduction by Lawrence Godkin, medico-legal inspections and post-mortem examinations are taken up by Dr. A. S. Briston. This is a chapter to be studied by everybody who is called in cases of sudden death.

The next section, and one of absorbing interest, treats of death in its medico-legal aspects, and is written by Dr. Francis A. Harris. These two sections should be studied together.

In this volume the medical jurisprudence of life insurance is dealt with *in extenso* by Dr. Brandreth Symonds, and an interesting chapter occurs on the legal relations of physicians and surgeons to their patients and to one another, written by William A. Purrington, the distinguished counsel of the Medical Society of the County of New York.

Many other interesting subjects are dealt with in this volume, but we cannot here mention them in detail.

The mechanical execution of the work is beyond criticism, and many illustrations add to its interest and contribute to its perfection.

A second volume, soon to be issued, will complete the work which is sold only by subscription.

Annual Report of the State Board of Charities for the Year 1893. Albany: James B. Lyon, State Printer. 1894.

This volume gives an interesting exhibit of the work of this important branch of the state government for the year 1893. Besides the official report of the board there are several excellent papers relating to charity economics. In one of these relating to child-saving work, by Hon. William P. Letchworth, we learn that the first institution in the state for the care of foundlings was established in Buffalo.

A considerable portion of the report relates to a consideration of the institutions conducting charitable and reform work in the eighth judicial district, also prepared by Mr. Letchworth. There are fifty-four institutions herein described, thirty-seven of which are classified under the head of miscellaneous and seventeen as medical charities. Thirty of the miscellaneous institutions are located in Buffalo, as are likewise fifteen of the medical charities.

This is one of the most useful reports published by the board, and deserves a careful examination by all philanthropists and others interested in charity work.

---

Transactions of the American Gynecological Society. Volume XIX. For the year 1894. Small 8vo, pp. xliii.—363. Philadelphia: Wm. J. Dornan, Printer. 1894.

The annual volume of this society always serves to enrich the literature of gynecic medicine. The present book is replete with excellent papers and good discussions. There are ninety-one active members of the society, fifty-seven of whom were present at the nineteenth annual meeting. This speaks well for the discipline of the organization and the interest taken in it by the Fellows.

Dr. J. C. Reeve, of Dayton, O., contributes an admirably written memorial of Dr. Alexander Dunlap, which deserves to be read by every American physician. It is accompanied by a fine photographic likeness of this veteran ovariotomist, which does ample justice to his striking features, and which brings out his strong character in ample manner.

Dr. Matthew D. Mann, of Buffalo, was elected president for the ensuing year, and the next place of meeting is Baltimore, on the fourth Tuesday of May, 1895.

---

Transactions of the Association of American Physicians. Ninth session, held at Washington, D. C., May 29, 30, 31, and June 1, 1894. Philadelphia: Printed for the Association. 1894.

The annual transactions of this association are always welcome. The annual address of the president, Dr. Reginal H. Fitz, of Boston, was entitled, The Rise and Fall of the Licensed Physician in Massachusetts, 1781—1860. In its concluding portion he says:

"Ten years of increasing tolerance destroyed seventy years of enthusiastic effort, devoted labor, tactful management and wise counsel in the public interest. The state revoked the control of medical practice and the people had been the sufferers. The history of Massachusetts in this respect is the history of the country. She was one of the last of the states to lay down the control and she will be one of the last to resume it."

The papers in this volume have been published in the magazines and have already become a part of the literature of medicine.

---

Transactions of the Medical and Chirurgical Faculty of the State of Maryland. Ninety-sixth annual session, held at Baltimore, Md., April, 1894, also semi-annual session, held at Annapolis, Md., November, 1893.

This volume grows thinner year by year. We speak, however, on this occasion with reference to its size, for though there are within its covers but two scientific papers, they furnish ample opportunity for study and reflection. The first is the president's address, by Dr. George H. Rohé, on the Extinction of Tuberculosis. This is a carefully written, scholarly paper and presents some of the most modern thought on the subject. The annual address by Dr. James T. Whittaker, of Cincinnati, O., has for its title, Predisposition to Phthisis. Some of the generally accepted opinions with reference to the supposed heredity of phthisis will here be found controverted.

---

### BOOKS RECEIVED.

The Proceedings of the Fourth Annual Meeting of the Association of Military Surgeons of the United States. Held at Washington, D. C., on May 1, 2 and 3, 1894. St. Louis: Buxton & Skinner Stationery Co. 1894.

Transactions of the Medical Society of the State of Pennsylvania, at its Forty-fourth Annual Session, held at Philadelphia, 1894. Volume XXV. Published by the society. Philadelphia: The Edwards & Docker Co., Printers. 1894.

A Dictionary of Medicine, including General Pathology, General Therapeutics, Hygiene and the Diseases of Women and Children, by various writers. Edited by Richard Quain, Bart., M. D., London, LL. D. Ed., F. R. S., President of the General Council of Medical Education; Member of the Senate of the University of London; Hon. M. D. Trinity College, Dublin; Hon. M. D. Royal University of Ireland; Fellow, late Vice-President, and Senior Censor of the Royal College of Physicians; Hon. Fellow of the Royal College of Physicians of Ireland; Physician Extraordinary to Her Majesty the Queen; Consulting Physician to the Hospital for Diseases of the Chest, Brompton, and to the Seamen's Hospital, Greenwich. Assisted by Frederick Thomas Robarts, M. D., London, B. Sc., and J. Mitchell Bruce, M. A., Aberdeen, M. D., London, with an American Appendix, by Samuel Treat Armstrong, M. D., Ph. D., Visiting Physician to the Harlem, Willard Parker and Riverside Hospitals, New York, etc. New edition, revised throughout and enlarged. Imperial 8vo, in two volumes, pp. 2566. Illustrated. New York: D. Appleton & Company. 1894.

Bureau of Education. Contributions to American Educational History. Edited by Herbert B. Adams. No. 14. The History of Education in Connecticut, by Bernard C. Stenier. A. M No. 15. The History of Education in Delaware, by Lyman P. Powell, A. B. No. 16. Higher Education in Tennessee, by Lucius Salisbury Merriam, Ph. D. No. 17. Higher Education in Iowa, by Leonard F. Parker. Washington: Government Printing Office. 1893.

The Johns Hopkins Hospital Reports. Report in Surgery II., Volume IV. No. 6. The results of operations for the cure of cancer of the breast performed at the Johns Hopkins Hospital from June, 1889, to January, 1894. By William S. Halsted, M. D., Professor of Surgery Johns Hopkins University; Surgeon-in-chief to the Johns Hopkins Hospital. Baltimore: The Johns Hopkins Press. 1894.

International Clinics. A Quarterly of Clinical Lectures on Medicine, Neurology, Pediatrics, Surgery, Genito-Urinary Surgery, Gynecology, Pharyngology, Rhinology, Ophthalmology, Laryngology, Obstetrics, Otology and Dermatology. By professors and lecturers in the leading medical colleges of the United States, Great Britain and Canada. Edited by Judson Daland, M. D. (Univ. of Penna.), Philadelphia, Instructor in Clinical Medicine and Lecturer on Physical Diagnosis in the University of Pennsylvania; Assistant Physician to the University Hospital; Physician to the Philadelphia Hospital and to the Rush Hospital for Consumption. J. Mitchell Bruce, M. D., F. R. C. P., London, England, Physician and Lecturer on Therapeutics at the Charing Cross Hospital. David W. Finlay, M. D., F. R. C. P., Aberdeen, Scotland, Professor of Practice of Medicine in the University of Aberdeen; Physician to, and Lecturer on, Clinical Medicine in the Aberdeen Royal Infirmary; Consulting Physician to the Royal Hospital for Diseases of the Chest, London. Volume III Fourth series. 1894. Royal 8vo, pp. xii.—363. Philadelphia: J. B. Lippincott Co. 1894.

Diagnosis, Differential Diagnosis and Treatment of Diseases of the Eye. By A. E. Adams, M. D., Instructor in Diseases of the Eye in the Post-Graduate Medical College; Assistant Surgeon to Manhattan Eye and Ear Hospital, New York; Ophthalmic Surgeon to St. Luke's Hospital, etc., etc. Duodecimo, pp. 94. New York: G. P. Putnam's Sons, 27 West Twenty-third street. 1894.

A Synopsis of the Practice of Medicine. For Practitioners and Students. By William Blair Stewart, A. M., M. D., Lecturer on Therapeutics; late Instructor on Practice of Medicine in the Medico Chirurgical College of Philadelphia; Demonstrator in the Philadelphia School of Anatomy, etc. One large 8vo volume, about 434 pages, cloth, $2.75. New York: E. B. Treat, 5 Cooper Union. 1894.

The Pocket Anatomist. By C. Henri Leonard, A. M., M. D., Professor of Gynecology, Detroit College of Medicine. Leather, 300 pages, 193 illustrations, post-paid $1.00. Detroit, Mich.: The Illustrated Medical Journal Co., Publishers. 1894.

A Manual of the Practice of Medicine, prepared especially for Students. By A. A. Stevens, A. M., M. D., Lecturer on Terminology and Instructor in Physical Diagnosis in the University of Pennsylvania; Demonstrator of Pathology in the Woman's College, Philadelphia; Physician to St. Mary's Hospital, etc., etc. Third edition, revised. Illustrated. Small 8vo, pp. xviii.—501. Price, $2.50. Philadelphia: W. B. Saunders, 925 Walnut street. 1894.

A Manual of Modern Surgery, General and Operative. By John Chalmers Da Costa, M. D., Demonstrator of Surgery, Jefferson Medical College, Philadelphia; Chief Assistant Surgeon Jefferson Medical College, etc. Saunders' New Aid Series. Small 8vo, pp. 809, with 188 illustrations in the text and thirteen full-page plates in colors and tints. Price, $2.50 net. Philadelphia: W. B. Saunders, 925 Walnut street. 1894.

Essentials of Diseases of the Skin, including the Syphilodermata. Arranged in the form of Questions and Answers prepared especially for Students of Medicine. By Henry W. Stelwagon, M. D., Ph. D., Clinical Professor of Dermatology in the Jefferson Medical College; Physician to the Department of Skin Diseases, Howard Hospital, etc., etc. Saunders' Question Compends No 11. Third edition, revised and enlarged, with seventy-one letter-press cuts and fifteen half-tone illustrations. Philadelphia: W. B. Saunders, 925 Walnut street. 1894.

Home Treatment for Catarrhs and Colds. A handy guide for the Prevention, Care and Treatment of Catarrhal Troubles, Cold in the Head, Sore Throat, Hay Fever, Hoarseness, Ear Affections, etc. Adapted for use in the household and for vocalists, clergymen, lawyers, actors, lecturers, etc. By Leonard A. Dessar, M. D., Visiting Laryngologist to St. Mark's Hospital and to Mount Sinai Hospital Dispensary, etc. Illustrated. New York: Home Series Publishing Co., P. O. Box 1406. 1894.

Landmarks in Gynecology. By Byron Robinson, B. S., M. D., Chicago, Illinois, Professor of Gynecology in the Chicago Post-Graduate School; Gynecologist to the Woman's Hospital, the Charity Hospital and the Post-Graduate Hospital. The Physicians' Leisure Library Series. In two volumes. Price, cloth, 50 cents; paper, 25 cents. Detroit, Mich.: George S. Davis. 1894.

Transactions of the Michigan State Medical Society for the year 1894. Volume XVIII. Detroit: Published by the Society. 1894.

Practical Urinalysis and Urinary Diagnosis: A Manual for the Use of Physicians, Surgeons and Students. By Charles W. Purdy, M. D., Queen's University; Fellow of the Royal College of Physicians and Surgeons, Kingston; Professor of Uronology and Urinary Diagnosis at the Chicago Post-Graduate Medical School; Author of Bright's Disease and Allied Affections of the Kidneys; also of Diabetes: Its Causes, Symptoms and Treatment. With numerous illustrations, including photo-engravings and colored plates. In one crown 8vo volume, 360 pages, in extra cloth, $2.50 net. Philadelphia: The F. A. Davis Co., 1914 and 1916 Cherry street. 1894.

Medical Jurisprudence, Forensic Medicine and Toxicology. By R. A. Witthaus, A. M., M. D., Professor of Chemistry, Physics and Hygiene, in the University of the City of New York, etc., etc., and

Tracy C. Becker, A. B., LL. B., Counselor-at-Law and Professor of Criminal Law and Medical Jurisprudence in the University of Buffalo. In four volumes. Volume II. Large 8vo, 751 pages, illustrated with wood-cuts and three plates, two of them in colors. Price, in muslin, $5.00 ; in brown sheep and in law style, $6.00 per volume. Sold by subscription only. New York : William Wood & Co. 1894.

Text-book of Hygiene : A Comprehensive Treatise on the Principles and Practice of Preventive Medicine from an American Standpoint. By George H. Rohé, M. D., Professor of Therapeutics, Hygiene and Mental Diseases, in the College of Physicians and Surgeons, Baltimore ; Superintendent of the Maryland Hospital for the Insane ; Member of the American Public Health Association ; Foreign Associate of the Société Française d'Hygiène, etc. Third edition, thoroughly revised and largely rewritten, with many illustrations and valuable tables. Royal 8vo, 553 pages. Cloth, $3.00 net. Philadelphia : The F. A. Davis Co., 1914 and 1916 Cherry street. 1894.

A Clinical Manual of Diseases of the Eye, including a Sketch of its Anatomy. By D. B. St. John Roosa, M. D., LL. D., Professor of Diseases of the Eye and Ear in the New York Post-Graduate Medical School and Hospital ; Surgeon to the Manhattan Eye and Ear Hospital ; formerly Professor of Diseases of the Eye in the University of the City of New York and in the University of Vermont ; Consulting Surgeon to the Brooklyn Eye and Ear Hospital ; President of the New York Academy of Medicine ; Honorary Member of the Medico-Chirurgical Society of Edinburgh ; Honorary Fellow of the Academy of Medicine of Havana, Cuba, etc., etc. Octavo, 650 pages, 178 engravings in the text, nearly all original, two full-page chromo-lithographic plates, and a full-page black plate. Bound in extra muslin at $5.50, and in sheep at $6.50. New York : William Wood & Co. 1894.

Essentials of Chemistry and Toxicology. For the Use of Students in Medicine. Twelfth edition. (Wood's Pocket Manuals.) By R. A. Witthaus, A. M., M. D., Professor of Medical Chemistry and Toxicology in the University of Vermont ; Member of the Chemical Societies of Paris and Berlin, etc. 32mo, 314 pages, muslin, red edge, price $1.00. New York : William Wood & Co. 1894.

